# Editorial: Mitochondrial Disorders: Biochemical and Molecular Basis of Disease

**DOI:** 10.3389/fgene.2021.769770

**Published:** 2021-11-19

**Authors:** Guilhian Leipnitz, Grant M. Hatch, Al-Walid Mohsen, Ronald J. A. Wanders

**Affiliations:** ^1^ Post-Graduation Program in Biological Sciences, Biochemistry, Department of Biochemistry, Universidade Federal do Rio Grande do Sul, Porto Alegre, Brazil; ^2^ Department of Pharmacology and Therapeutics, University of Manitoba, Winnipeg, MB, Canada; ^3^ Division of Genetic and Genomic Medicine, Department of Pediatrics, School of Medicine, University of Pittsburgh, Pittsburgh, PA, United States; ^4^ Laboratory Genetic Metabolic Diseases, Departments Pediatrics, Emma Children Hospital and Clinical Chemistry, Amsterdam University Medical Center, Amsterdam, Netherlands

**Keywords:** mitochondrial disorders, case report, treatment, pathophysiology, reaction mechanism

Mitochondria are dynamic, double-membrane organelles that play an essential function in cellular energy metabolism, in which the citric acid cycle, fatty acid β-oxidation, and oxidative phosphorylation act in concert to generate most of the ATP in cells. However, in the last few decades, mitochondria have been reported to execute and regulate many other functions, including ATP production, metabolism of amino acids, lipids and nucleotides, iron-sulfur cluster synthesis, calcium homeostasis, and programmed cell death. Healthy mitochondria take up calcium not only to regulate their own intrinsic metabolism and stimulate the production of ATP, but also to buffer cytosolic calcium rises that occur during normal cell functioning (Dard et al., 2020). However, calcium influx beyond the buffering capacity of mitochondria, as well as reactive oxygen overproduction, trigger mitochondrial permeability transition that may cause apoptosis (Vercesi et al., 2018).

For maintenance of normal mitochondrial functionality, this organelle depends on a delicate balance between fission and fusion (mitochondrial dynamics). The fusion and fission cycle consists, respectively, of the formation of an elongated organelle by fusing two or more mitochondria and by the division of one mitochondrion into two daughter mitochondria (Tilokani et al., 2018). While mitofusins 1 and 2 and optic atrophy 1 protein mediate fusion of the outer and inner mitochondrial membrane, correspondingly, dynamin-related protein 1 is considered a crucial player in the fission process, forming oligomers around mitochondria to constrict them (Tilokani et al., 2018).

The pivotal role of mitochondria for cellular survival is highlighted by the variety of diseases that are associated with mitochondrial dysfunction. In this sense, mitochondrial inherited genetic disorders, which may be caused either by mutations in nuclear genes or mitochondrial DNA encoding mitochondrial proteins, usually affect multiple tissues, typically those which are highly dependent on aerobic metabolism (Gorman et al., 2016; Ferreira and van Karnebeek, 2019; Wajner, 2019). While primary defects are characterized by disturbances in oxidative phosphorylation and other energy generating pathways, secondary mitochondrial diseases result from the toxic influences of endogenous metabolites to the mitochondria and/or cell physiology in general. In this topic “Mitochondrial Disorders: Biochemical and Molecular Basis of Disease”, we provide a collection of nine articles covering diverse aspects of primary and secondary mitochondrial disorders.

The pathophysiology of mitochondrial disorders is the main theme of two articles. Amaral and Wajner review the toxic effects of fatty acids accumulating in fatty acid oxidation disorders causing impairment of mitochondrial bioenergetics and calcium homeostasis and inducing permeability transition pore opening. The group of Dr. Schwarz (Mellis et al.) shows alterations of mitochondrial dynamics in embryonic fibroblasts prepared from *Suox* ^*−/−*^ mice (isolated sulfite oxidase deficiency model) and fibroblasts from patients with molybdenum cofactor deficiency (*MOCS1A, MOCCS1B, MOCS2*, and *GPHN* genes), indicating that disturbances in the mitochondrial network are involved in the pathophysiology of these disorders.

The elucidation of the reaction mechanism of mitochondrial enzymes may help to understand the biochemical abnormalities associated with mitochondrial disorders. In this regard, the article by Leung et al. provides evidence that glycine cleavage system H protein (GCSH) has an additional role beyond being a component of the glycine cleavage system, a protein complex that decarboxylates glycine. The study demonstrates that loss of GCSH causes embryonic death of homozygous *Gcsh* null mice, unlike animals with a deficiency of the other proteins belonging to the glycine cleavage system, which are compatible with embryonic survival. Furthermore, Cronan reviews the reaction mechanism of lipoic acid metabolism enzymes with the aim to better understand the biochemistry and physiology of mitochondrial disorders that affect the assembly of this coenzyme on its cognate enzyme proteins.

In addition, different models for mitochondrial disease research are reviewed by the group of Dr. Sánchez-Alcázar (Povea-Cabello et al.), with emphasis on direct cellular reprogramming. Another article by Schlieben and Prokisch (2020) gives an overview of the mitochondrial disease associated genes, with focus on the classification of these disorders according to the functional roles of deficient proteins, with implications for diagnosis. This topic also includes a perspective article on the outcome of triheptanoin in treating long chain fatty acid oxidation disorders, with special emphasis on the cardiomyopathy and hypoglycemia observed in affected patients (Sklirou et al.).

Finally, two articles present case reports of primary mitochondrial disorders. Kušíková et al. (2021) give a brief review on the mitochondrial disorder caused by mutations in *VARS2*, which encodes the mitochondrial valyl-tRNA synthetase, and presents a patient with a new missense biallelic variant leading to a novel phenotypic presentation. Moreover, (Yan et al.) report the first three Chinese cases of combined oxidative phosphorylation deficiency 23 (COXPD23), which is caused by mutations in *GTPBP3*, as well as genotype-phenotype correlations of these patients.

In conclusion, this topic provides a valuable collection of articles revealing important aspects of the genetic and biochemical basis and pathophysiology of mitochondrial disorders. While most articles emphasize bioenergetic dysfunction as a fundamental mechanism in mitochondrial disorders, this topic also explores the involvement of other mitochondrial functions and processes that are impaired in these pathologies, such as calcium homeostasis, mitochondrial dynamics, and permeability transition. We hope that the articles presented here stimulate future studies not only focused on the expansion of current knowledge but also on the development of novel adjuvant therapeutic strategies with the aim to provide new treatment options and/or to prevent these devastating conditions.
